# Unprepared at Enrollment: Assessing the Financial Literacy and Employee Benefit Selection Preparedness of Graduating Medical Students

**DOI:** 10.7759/cureus.90949

**Published:** 2025-08-25

**Authors:** Kareem Joudi, Simon Tacvorian, Shyamal R Asher

**Affiliations:** 1 Anesthesiology, Rhode Island Hospital/The Warren Alpert Medical School of Brown University, Providence, USA

**Keywords:** disability insurance, employer benefits, fourth-year medical students, health insurance, open enrollment, physician financial literacy, retirement planning

## Abstract

Introduction

As graduating medical students transition to residency, they are required to make critical financial decisions regarding employer-sponsored benefits such as health insurance, retirement plans, and disability coverage. Despite the significant impact of these decisions, many students receive little to no formal financial education during medical school and thus are unprepared to manage and navigate these personal finance decisions. This study aims to evaluate graduating medical students' baseline knowledge in key financial domains regarding employer-sponsored benefits.

Methods

We conducted a prospective, single-institution study of fourth-year medical students at The Warren Alpert Medical School of Brown University during the Internship Preparatory Course over two years. Students were invited to complete an anonymous, voluntary survey consisting of 15 multiple-choice questions assessing basic knowledge across three domains: health insurance, retirement plans, and disability insurance. Responses were categorized as correct, incorrect, or “don’t know” to prevent respondents from attempting to guess answers. Survey results were stratified by prior participation in open enrollment or ownership of employer-sponsored retirement accounts. Two-sample t-tests compared performance between groups.

Results

Out of 129 students attending the workshop, 75 completed the survey (response rate: 58%). The median age was 27 years (±2); 14.7% had previously participated in an open enrollment process, and 29.3% had prior experience with an employer-sponsored retirement account. Overall, the average percent of correct responses was 11.4% and incorrect responses 26.4%, and the “don’t know” option was selected in 62.2% of questions. Domain-specific performance showed 15.7% correct in health insurance, 17.9% in retirement, and 0% in disability insurance, with nearly 70% of disability-related responses being selected as “don’t know.” No students answered any disability insurance questions correctly. Students with prior open enrollment experience or retirement accounts performed slightly better (average correct: 12.7% vs. 10.9% and 12.7% vs. 10.6%, respectively), but these differences were not statistically significant.

Conclusion

This study reveals critical deficiencies in financial literacy among graduating medical students, particularly in selecting and understanding employer-sponsored benefit options that they will encounter in residency. Despite modest differences based on prior exposure, knowledge gaps remained profound across all domains. These findings support a growing body of literature advocating for the integration of structured, targeted financial education into the medical school curriculum. Equipping future physicians with essential financial knowledge prior to entering residency may reduce costly mistakes that compound over time, improve financial literacy and well-being, and promote more informed benefit selection early in their careers.

## Introduction

Medical students devote many years to mastering clinical skills and medical knowledge, yet many complete their training with minimal exposure to essential financial literacy concepts. As residency begins, these graduates are required to make critical decisions regarding employer-sponsored benefits such as when selecting health insurance plans, retirement accounts, and savings options. Studies have shown that over 90% of graduating medical students would desire more education on financial topics as part of their medical education [1]. It has been reported that more than 90% of resident physicians feel that they are unable to handle their finances [2]. Trainees with poor financial literacy are less likely to plan for retirement and are more likely to use high-cost means of borrowing [3-5]. Prior studies have demonstrated that residents and fellows score about 50% in personal finance literacy tests [6,7]. As medical students are graduating with an average of approximately $235,000 in debt [8], and many students are earning a salary for the first time in their lives, financial literacy becomes that much more important to set students up for financial success throughout their career. Prior studies have shown improvement in financial literacy and improved confidence in financial knowledge in medical students following participation in a financial workshop [9].

The limited knowledge about basic personal finance knowledge including savings and retirement is consistent with the fact that medical students receive minimal personal finance education in their curriculum. In spite of this poor preparation, at the start of residency, new residents are required to engage with employer benefits within the first month, where they likely first encounter topics such as retirement accounts, health insurance, and various types of disability insurance options. These newly minted residents are given a crash course on the topic, usually from a human resources representative. This is usually in the context of orientation to their clinical duties, which get more importance during the orientation period.

Prior behavioral science studies have shown that once employees sign up for benefits, they are likely to keep the same selection for the duration of their employment. This is referred to as the status quo bias. In a classic study, Samuelson and Zeckhauser showed minimal changes in healthcare and retirement benefits within employees of a large academic institution [10]. Furthermore, the choices of newly enrolled employees differed significantly from continuing plan employees. This pattern is interpreted as status quo bias, as continuing employees stick with their initial health plans despite the availability of new and better choices that are selected by new employees who are free of this bias [10]. These effects are likely magnified for medical residents for the duration of their training and perhaps early attending years due to the demanding work hours and steep learning curve within their medical practice.

In this context, the need for targeted financial education for graduating medical students is clear. This prospective, single-institution study aimed to evaluate the baseline knowledge of graduating medical students in key financial domains regarding employer-sponsored benefits in preparation for employer-sponsored benefit selection during residency.

## Materials and methods

The Rhode Island Hospital Institutional Review Board determined that this study is exempt from Human Subjects Research under 45 Code of Federal Regulations 46.104(d) requirements and does not require consent documentation (IRB# 2140702). Participants received an introductory letter describing the nature of the study and their completion of the survey indicating their willingness to participate in the study.

Study design and participants

A prospective, single-institution, needs assessment study was conducted during the Spring 2023 and 2024 semester at The Warren Alpert Medical School of Brown University. All fourth-year medical students scheduled to graduate that spring were invited to attend a basic personal finance workshop as part of the Internship Preparatory Course (IPC). The students self-select five out of 20 workshops to earn credit for the IPC, which is a requirement for graduation. At the start of the workshop, the students were asked to voluntarily participate in this study by completing a survey questionnaire at the start of the session. Completion of the survey was voluntary and anonymous and had no bearing on participation in the remainder of the workshop. No personal identifying information was collected.

Survey

**Figure 1:**
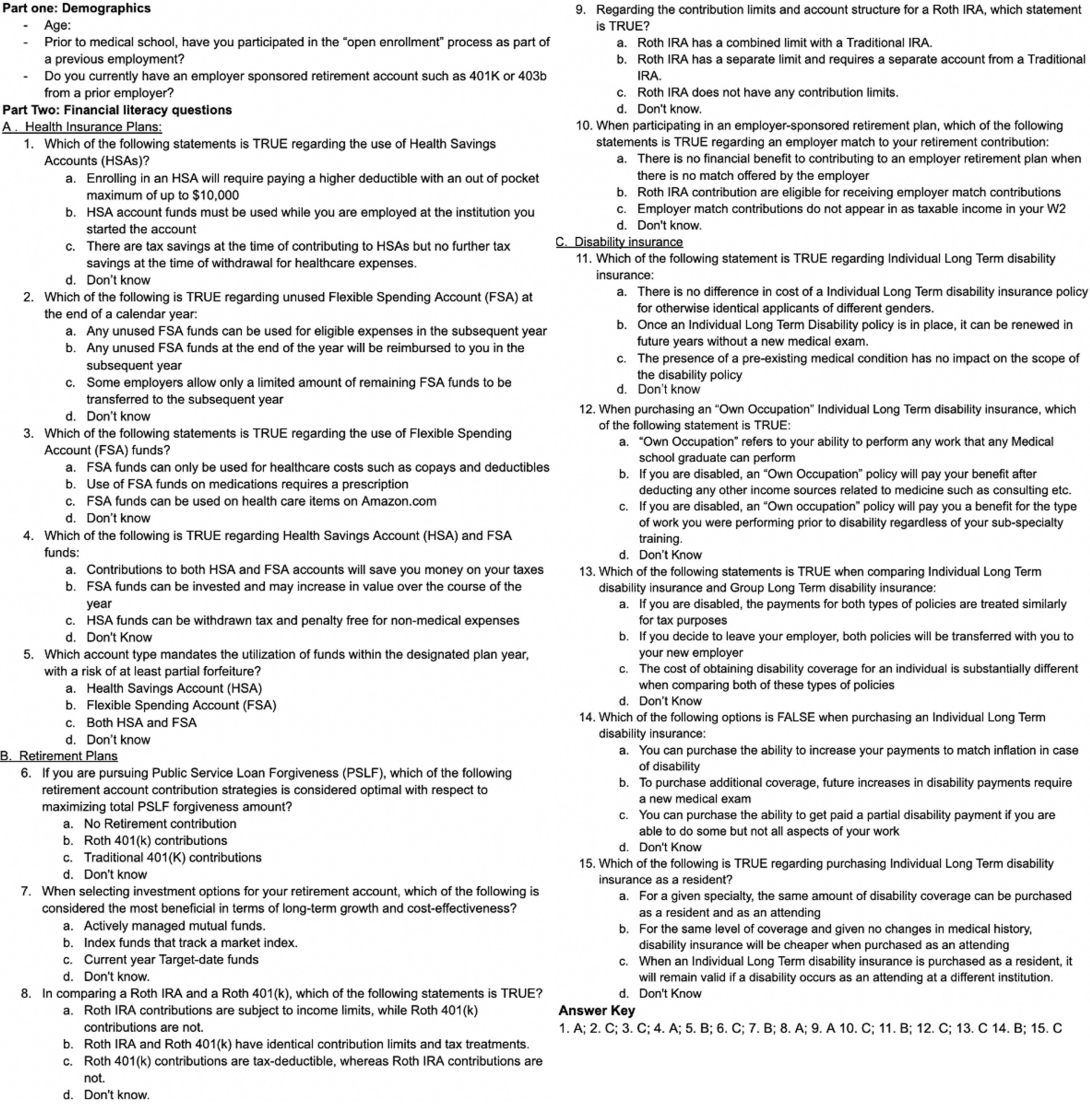
Survey questionnaire

The survey consisted of two parts: the first part obtained basic baseline characteristics of the students, while the second part consisted of a total of 15 questions regarding standard employer-sponsored benefits. Questions were developed and refined by this team of authors including a medical student, resident physician, and attending physician with experience in personal finance topics. Questions were formed surrounding common options training physicians are given when choosing from various employer-sponsored benefit options. There were five questions on details of health insurance plans, five questions on basic knowledge of retirement plans, and five questions on the selection of disability insurance options. Each question had four potential answer choices, three being potential answer choices to the question and one “I don’t know” option to reduce the incidence of guessing by the participants. All students present for the workshop were invited to participate via a Google Form (Google, Mountain View, CA, US) survey accessed via a QR code, ensuring voluntary participation and maintaining anonymity. Informed consent was obtained from all participants. The survey is presented in Figure 1.

After the completion of the survey, all participants attended a three-hour interactive workshop hosted by one of the authors (SA) on basic personal finance covering a variety of topics including student loan management, saving for retirement including retirement plan overview, basics principles of investing and choosing investments, budgeting and saving strategies, disability insurance, and basic behavioral economics as applied to personal finance.

Survey responses were analyzed using descriptive statistics. For each respondent, the percentage of correct, incorrect, and "don't know" responses was calculated overall and within each content category (health insurance, retirement plans, and disability insurance). For subgroup analyses, respondents were stratified by prior participation in an employer open enrollment process and by prior ownership of an employer-sponsored retirement account.

To compare knowledge performance between groups (e.g., those with vs. without prior open enrollment experience), the proportion of correct responses per respondent was compared using independent two-sample t-tests. This approach was used to evaluate differences in overall percent correct, as well as percent correct within each content category. For the disability insurance category, p-values were not computed due to the absence of correct responses in all groups. Statistical significance was set at a two-sided alpha of 0.05. Analyses were conducted using R (version 4.5.0, R Core Team 2024, https://www.r-project.org/).

## Results

Participant demographics

A total of 129 medical students over the two years attended the personal finance workshop, with 75 students completing the employee benefits questionnaire (58% response rate). All surveys submitted and collected were fully completed with no missing responses. The median age of respondents was 27 years (±2), with 14.7% (n = 11) having participated in an open enrollment process prior to medical school and 29.3% (n = 22) reporting participation in a previous employer-sponsored retirement account such as a 401(k) or 403(b). This information is summarized below in Table 1.

**Table 1:** Demographics of study participants

	Participants (n = 75)
Median age ± SD (years)	27 ± 2
Previous employer-sponsored retirement account (n, %)	22 (29.3%)
Participation in open enrollment before medical school	11 (14.7%)

Survey performance

Overall, students demonstrated significant gaps in knowledge related to employee benefits relevant to residency and early career employment for graduating medical students. The average percent of correct responses across all questions and participants was 11.4%, with an average of 26.4% incorrect responses and 62.2% “don’t know” responses. No participant answered all questions correctly, and the most common response for the majority of questions was “don’t know.”

Survey questions were grouped into three content domains: health insurance (questions 1 through 5), retirement plans (questions 6 through 10), and disability insurance (questions 11 through 15). The average percent correct for health insurance questions was 15.7%, for retirement plans was 17.9%, and for disability insurance was 0.0%. Notably, the vast majority of students selected “don’t know” for questions regarding disability insurance (average 69.3%), and no respondent answered any disability insurance question correctly.

**Figure 2:**
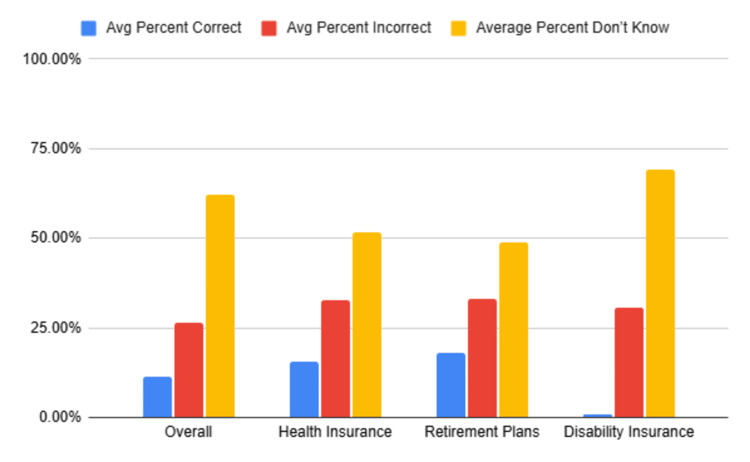
Overall survey questionnaire results and breakdown of results in the content domains of health insurance, retirement plans, and disability insurance

These results are summarized in Table 2 and Figure 2.

**Table 2:** Overall survey questionnaire results and breakdown of results in the content domains of health insurance, retirement plans, and disability insurance

Survey questions	Average percent correct	Average percent incorrect	Average percent “don’t know”
Overall (15 Qs)	11.4%	26.4%	62.2%
Health insurance (5Qs)	15.7%	32.8%	51.6%
Retirement plans (5Qs)	17.9%	33.%	49.0%
Disability insurance (5Qs)	0%	30.7%	69.3%

Performance by prior experience

Students with prior experience participating in an open enrollment process or with a previous employer-sponsored retirement account performed modestly better overall, but differences were not statistically significant. Specifically, respondents who had participated in open enrollment scored an average of 12.7% correct versus 10.9% for those without such experience (p = 0.55). Similarly, those with a prior employer-sponsored retirement account averaged 12.7% correct, compared to 10.6% for those without (p = 0.41). No significant differences were observed between groups for health insurance or retirement plan knowledge, and no group answered any disability insurance question correctly. Subsequently, all p-values for disability insurance were non-computable due to a universal 0% correct. Table 3 compares the results of participants with and without previous open enrollment experience. Table 4 compares the results of participants with and without previous employer account experience.

**Table 3:** Survey questionnaire results of participants stratified by with and without previous open enrollment experience

Group	N	% correct overall (p = 0.55)	% correct health insurance (p = 0.41)	% correct retirement plans (p = 0.95)	% correct disability insurance
Previous open enrollment experience	11	12.7%	20.0%	18.2%	0%
No previous open enrollment experience	64	10.9%	15.0%	17.8%	0%

**Table 4:** Survey questionnaire results of participants stratified by with and without previous employer account experience

Group	N	% correct overall (p = 0.41)	% correct health insurance (p = 0.21)	% correct retirement plans (p = 0.92)	% correct disability insurance
Previous employer account	22	12.7%	20.0%	19.2%	0%
No previous employer account	53	10.6%	14.0%	17.7%	0%

## Discussion

The results of this study demonstrate a significant deficit in financial knowledge regarding employer-sponsored benefits among graduating medical students. On average, respondents answered only 11.4% of questions correctly, and the majority of responses, 62.2%, were “don’t know,” reinforcing previously documented deficits [6,7] and highlighting the urgent need for structured education in the medical curriculum. In a single-institution workshop study at Brown University, senior medical students demonstrated only 54% correct answers on a personal finance quiz at baseline, improving to 62% post-intervention, particularly in areas like retirement savings and insurance [9]. A recent systematic review of 38 studies involving medical students and early-career healthcare professionals confirmed that financial literacy is generally suboptimal, especially on topics such as insurance, retirement planning, and benefits [11]. Another international study demonstrated that 75% of medical students failed a financial knowledge module and nearly all students showed major gaps in benefits-related domains, including tax-advantaged accounts and insurance coverage [12]. These findings are consistent across diverse settings and question formats, especially for knowledge of employer benefits like health savings accounts (HSAs), flexible spending accounts (FSAs), and disability insurance. Together, this literature and our study reveal a persistent and pervasive knowledge gap among graduating medical students in critical financial domains that directly impact their transition to residency and professional practice.

Performance was particularly poor in the area of disability insurance, where no student answered any of the questions correctly and nearly 70% selected “don’t know.” Disability insurance is a form of income protection that replaces a portion of your income if injury, illness, or disability prevents you from performing your occupation. For physicians, whose primary asset is their ability to earn a high income, this protection is extremely important. According to the Council for Disability Awareness, roughly 30% of working-age adults will experience a disability lasting three months or longer before age 65; about one in seven may encounter a disability of five years or more [13]. For doctors specifically, many sources emphasize the value of an “own‑occupation” policy definition, which pays benefits if one is unable to practice in their medical specialty even if they can perform some other medical job. Industry data estimate that about one in seven physicians will file a disability insurance claim sometime during their careers [14]. Despite these clear benefits and documented usage among physicians, our students demonstrated negligible knowledge in this subject, with 0% correct responses and a predominance of “don’t know” answers. This shows that although disability insurance is widely recommended and frequently utilized by physicians to safeguard their future earnings, graduating students appear unprepared despite its importance. Understanding this disparity in lack of preparedness yet great importance reinforces the need for integrating structured education about benefits like disability coverage into medical school curricula to ensure that future physicians can make informed decisions to protect both their income and their long-term financial resilience.

Although performance on retirement and health insurance questions was somewhat better, overall knowledge remained low, and most students did not demonstrate readiness to navigate real-world employer-sponsored benefit decisions. Retirement planning involves intentionally saving and investing income to support financial independence in later life. For physicians, this process is particularly critical due to prolonged training, delayed earnings, and substantial student debt upon graduation. In a recent survey by the Teachers Insurance and Annuity Association of America (TIAA) Institute, 34% of healthcare professionals lacked confidence in being on track, and many were unclear about proper investment strategies [15]. Without early and structured financial planning during residency and early career stages, physicians may miss key opportunities such as employer matching or tax-advantaged accounts that can significantly impact long-term wealth accumulation.

In our study, average performance on retirement-related questions was only 17.9% correct, with nearly half of the responses marked “don’t know.” This low level of proficiency supports the broader concern that while many trainees participate in employer retirement plans, understanding how to leverage them effectively is lacking. Our findings add to the growing call for structured retirement education within the medical curriculum to ensure students are not simply enrolling in plans but truly understanding and optimizing them.

For medical students and residents preparing to select health plans during residency open enrollment, understanding distinctions in plan options is essential. Knowledge gaps in these areas can result in overspending, inefficient tax advantages, or choosing suboptimal plans during critical financial transitions. Fourth-year medical students often demonstrate poor proficiency in these skills. In a 2022 survey of US senior medical students, only 28.4% could correctly determine out-of-pocket costs in clinical scenarios, and average confidence scores on the Health Insurance Literacy Measure used were low, with mean confidence scores near 2.1 on a 1-4 scale (where 4 indicates maximum confidence) [16].

In our survey, health insurance-related questions had an average of 15.7% correct answers, with over 51% responding “don’t know.” This demonstrates how students lack foundational knowledge on health insurance plans, which is likely to limit their ability to make fiscally sound plan choices in their transition to residency. These results reinforce the urgent need for targeted education on health benefits selection during medical training.

Notably, students with prior exposure to employer-sponsored benefits, such as open enrollment processes or retirement accounts, had a trend toward better performance overall; however, these results were not statistically significant. This may suggest that passive exposure to financial decisions in workplace settings may not be sufficient to impart lasting understanding or confidence. Instead, a more structured, educational approach may be required to address these persistent gaps in knowledge.

This study is subject to several limitations. The survey was conducted at a single institution with a limited sample size and may not generalize to all medical student populations. Additionally, self-selection bias is possible, as students with a greater interest in financial topics may have been more likely to participate in the elective workshop. Similarly, response bias may be present with the 58% response rate among workshop attendees. The survey design focused on selected domains; other important areas of financial literacy, such as tax planning and contract negotiation, were not assessed. Furthermore, while the survey tool was reviewed by a multidisciplinary team with financial experience, the tool did not undergo further formal validation or reliability testing. This topic would benefit from future research conducted by more institutions and including larger participant populations, as well as including pre-/post-surveys to assess various education format efficacies, as this was not performed in this study.

## Conclusions

In summary, graduating medical students in this sample demonstrated low levels of financial knowledge across key areas relevant to the selection of employer-sponsored benefits that accompany the transition to residency. The high proportion of “don’t know” responses and lack of correct answers in critical areas such as disability insurance illustrate a deficiency in financial knowledge in this study’s population. Further research confirming this study’s results in various populations will help highlight the need for structured financial education within the medical school curriculum. Interventions that go beyond passive exposure and instead provide direct, practical instruction on health insurance, retirement planning, and disability insurance may be warranted. Preparing future physicians with essential financial knowledge will better position them to make informed decisions, protect their financial well-being, and focus on their clinical training during the pivotal years of residency.
